# Validating reference microRNAs for normalizing qRT-PCR data in bovine oocytes and preimplantation embryos

**DOI:** 10.1186/s12861-015-0075-8

**Published:** 2015-06-12

**Authors:** Mahdi Mahdipour, Helena T.A. van Tol, Tom A.E. Stout, Bernard A.J. Roelen

**Affiliations:** Department of Farm Animal Health, Faculty of Veterinary Medicine, Utrecht University, Yalelaan 104, 3584 CM Utrecht, The Netherlands; Department of Equine Sciences, Faculty of Veterinary Medicine, Utrecht University, Yalelaan 104, 3584 CM Utrecht, The Netherlands

**Keywords:** Reference miRNA, Bovine, Porcine, Oocyte, Embryo, qRT-PCR

## Abstract

**Background:**

MicroRNAs (miRNAs) are small noncoding RNAs that act as post-transcriptional regulators of gene targets. Accurate quantification of miRNA expression using validated internal controls should aid in the understanding of their role in epigenetic modification of genome function. To date, most studies that have examined miRNA expression levels have used the global mean expression of all expressed genes or the expression of reference mRNAs or nuclear RNAs for normalization.

**Results:**

We analyzed the suitability of a number of miRNAs as potential expression normalizers in bovine oocytes and early embryos, and porcine oocytes. The stages examined were bovine oocytes at the germinal vesicle (GV) and metaphase II stages, bovine zygotes, 2, 4 and 8 cell embryos, morulae and blastocysts, as well as porcine cumulus oocyte complexes, GV, metaphase I and II oocytes. qRT-PCR was performed to quantify expression of miR-93, miR-103, miR-26a, miR-191, miR-23b, Let-7a and U6 for bovine samples and miR-21, miR-26a, miR-93, miR-103, miR-148a, miR-182 and miR-191 for porcine oocytes. The average starting material for each sample was determined using specific standard curves for each primer set. Subsequently, geNorm and BestKeeper software were used to identify a set of stably expressed miRNAs. Stepwise removal to determine the optimum number of reference miRNAs identified miR-93 and miR-103 as the most stably expressed in bovine samples and miR-26a, miR-191 and miR-93 in porcine samples.

**Conclusions:**

The combination of miR-93 and miR-103 is optimal for normalizing miRNA expression for qPCR experiments on bovine oocytes and preimplantation embryos; the preferred combination for porcine oocytes is miR-26a, miR-191 and miR-93.

**Electronic supplementary material:**

The online version of this article (doi:10.1186/s12861-015-0075-8) contains supplementary material, which is available to authorized users.

## Background

During mammalian embryogenesis, primordial germ cells migrate to the genital ridge, and the somatic cells in these structures direct germ cell development [1]. In females, after several mitotic divisions the germ cells develop into primary oocytes, enter meiosis and then arrest at prophase I of the first meiotic division. Oocytes of most mammalian species resume meiosis only shortly before ovulation, and arrest again at the metaphase II stage until activated by sperm penetration at fertilization [2]. After fertilization, the zygote embarks on a series of cleavage divisions to form a multicellular embryo. Depending on the species, the embryonic genome is switched on somewhere between the 2- and 8-cell stages [3-6].

MicroRNAs (miRNAs) are small (19–24 nucleotides) non-coding RNAs, that have been identified in plants, animals and viruses and are involved in the regulation of gene expression at both transcriptional and post-transcriptional levels. They bind to the 3’-UTR of their target mRNA and either inhibit translation or induce degradation of that mRNA [7-10]. miRNAs are key components of gene regulation and are involved in various biological processes such as control of the cell cycle, apoptosis [11, 12], and regulation of developmental processes and embryogenesis [13, 14]. Aberrant expression of miRNAs can lead to various disease states, including tumor formation [15-17]. Several studies have demonstrated the presence of miRNAs in oocytes and established their importance during oocyte maturation and early embryo development [18-23].

One issue that has not yet been fully addressed however, is the accurate quantification of miRNA expression levels. Quantitative reverse transcription PCR (qRT-PCR) is a sensitive and relatively rapid method to examine gene expression levels in small numbers of cells [24]. In order to accurately determine gene expression levels by qRT-PCR, however, it is important to correct for factors that could influence starting or final RNA levels such as differences in the amount or nature of starting material, and the methods of RNA isolation and cDNA synthesis. In addition minimizing technical variation is essential to identifying real expression differences between samples [25]. Thus the accuracy of expression data, and any conclusion based on expression patterns, is highly dependent on valid normalization strategies [26].

Gene expression levels can be normalized using stably expressed genes, referred to as reference genes. These genes are generally selected on the basis of having the least variation in expression across the tissues of interest. Although genes coding for basic metabolic processes, such as that coding for glyceraldehyde-3 phosphate dehydrogenase (GAPDH), are constitutively expressed in many cells and have been used as reference genes for a variety of cell and tissue types in various species, their selection does require validation by examining and determining the most stable potential reference genes within the cells, tissue or samples of interest [25, 27].

Although several miRNAs and nuclear RNAs have been used for normalizing levels of miRNA expression determined by qRT-PCR, there are no convincing data demonstrating their stable expression in bovine oocytes and embryos. In the present study, we examined the expression of various miRNAs and a nuclear RNA in order to identify the most stably expressed miRNAs in bovine oocytes and pre-implantation embryos, and porcine oocytes of different maturation stages.

## Methods

### Oocyte collection, maturation and fertilization

Bovine ovaries were collected from a slaughterhouse, and transported in a polystyrene box; they arrived at the laboratory within 2 h after slaughter. After washing, the ovaries were transferred to a flask containing 0.9 % NaCl supplemented with penicillin/streptomycin (1 ml/L) and maintained at 30 °C in a water bath. Cumulus oocyte complexes (COCs) were collected by aspirating the contents of 2–8 mm diameter follicles, and selected under a microscope based on the presence of a multilayered cumulus complex. Selected COCs were matured and fertilized as described previously [28]. In short, oocytes were cultured in maturation medium and fertilized after 23–24 h of maturation with 1 × 10^6^/ml sperm from a bull of proven fertility. The cumulus cells were removed by vortexing 18–22 h after sperm addition, and the oocytes were transferred to pre-equilibrated synthetic oviductal fluid (SOF). Presumptive zygotes were cultured in a humidified incubator at 38.5 °C, 5 % CO_2_ and 7 % O_2_. At day 5 of culture, cleaved embryos were transferred to fresh SOF and cultured until day 8. Oocytes and embryos were collected at 0 h (germinal vesicle), 23 h (metaphase II) of maturation, 20, 32, 38, 56 h post fertilization (for zygote, 2, 4 and 8 cell embryo), day 5 (morula) and day 8 (blastocyst) then stored at −80 °C until small RNA extraction. Only those oocytes and embryos were collected that were indeed at the correct developmental stages as presence of arrested cells or embryos could alter the presence of miRNAs. Porcine ovaries were processed similarly to bovine ovaries. COCs were recovered by aspiration from follicles with a diameter of 2–5 mm. Selected COCs were matured as described previously [29]. Cumulus cells were removed after 24 or 48 h of maturation to yield MI and MII oocytes respectively.

### miRNA isolation and cDNA synthesis

Isolation of total miRNA was performed using the miRCURY RNA Isolation Kit (300110; Exiqon, Vedbaek, Denmark), with some modifications. Briefly, oocytes and embryos (20 per group) were lysed in 350 μl lysis buffer, mixed with 200 μl of 100 % ethanol and pipetted directly onto an RNA-binding column. After washing, miRNA was eluted using 50 μl elution buffer followed by a second elution with 50 μl of RNAse-free water. The eluent was concentrated using the RNeasy MinElute Cleanup Kit (74204, Qiagen, Valencia, CA, USA). Reverse transcription (RT) was performed using a miRCURY LNA, Universal cDNA Synthesis Kit II, (203301, Exiqon) in a total volume of 20 μl made up of 10 μl sample RNA, 4 μl 5x buffer, 2 μl RNAse-free water, 2 μl RNA spike (UniSp6) and 2 μl enzyme mix. The mixture was incubated for 1 h at 42 °C, followed by 5 min at 80 °C before storage at −20 °C.

### Quantitative RT-PCR

Quantitative RT-PCR was performed on three independent cDNA samples in duplicate. Samples were quantified simultaneously in one run on a 96-well plate using a real-time PCR detection system (MyIQ Single-color Real-Time PCR Detection System; Bio-Rad Laboratories, Hercules, CA, USA). Standard curves were made from serial dilutions of cDNA. The qRT-PCR reaction mixture (15 μl) contained 1 μl cDNA, 7.5 μl IQ™ Sybr® Green Supermix (Bio-Rad Laboratories), 1.5 μl primer mix and 5 μl RNAse-free water. Initial denaturation took place at 95 °C for 10 min, followed by 45 cycles each consisting of 10 s at 95 °C and 60 s at 60 °C (ramp rate of 1.6 °C/s). Melting curves were plotted at the end of each cycle series to verify the purity of the products. Progressive dilutions of cDNA from 450 bovine oocytes and 415 porcine oocytes were used to determine primer efficiencies and to generate the standard curves for each primer pair. The relative starting quantity for each experimental sample was calculated based on the standard curve made for each primer pair. Primers used for qRT-PCR were designed and obtained through the Exiqon website (http://www.exiqon.com).

### Statistical analysis

During qPCR amplification, amplification curves were generated for each sample, and subsequently a quantification cycle (Cq) value was calculated for each amplicon. Relative expression was calculated using each amplicon’s specific standard curve. These values were assessed using geNorm Version 3.5 [25] and BestKeeper Version 1 [30] to investigate expression stability. Using the geNorm software, the stability value (M) based on the average pairwise variation between all studied genes was calculated, whereas for BestKeeper a pair-wise correlation analysis of all pairs of candidate genes was determined and geometric expression means were calculated.

Results are presented in bar graphs as means ± standard error. Differences of miRNA expression in different samples were tested by ANOVA with a post hoc Fisher’s Least Significant Difference (LSD) test. A probability (P) below 0.05 was considered significant.

## Results

### Expression of candidate housekeeping miRNAs

The miRNAs examined in this study were selected primarily because their use and expression has been described in other species or tissues, in which they had relatively stable expression levels [26, 31-38]. Alternatively, their in-silico identified messenger RNA targets have important roles in general cell function such as RNA transcription suggesting that they could potentially act as reference miRNAs for normalizing expression data. In bovine samples, the expression of miR-23b, miR-26a, miR-93, miR-103, miR-191, Let-7a and the nuclear RNA U6 was examined. In porcine samples, the miRNAs identified as fluctuating least in bovine samples, namely miR-26a, miR-93, miR-103 and miR-191 were examined together with miR-21, miR-148a, and miR-182.

Identification of putative messenger RNA targets for miR-21, miR-23, miR-148a and miR-182 using gene ontology analysis suggested that these miRNAs may play important roles in regulation of cell development, morphogenesis, differentiation and apoptosis (data not shown).

The average amplification efficiency of the candidate reference miRNAs was 91.7 % (±7.7) with an average coefficient of determination (r^2^) of 0.994 (±0.005). Single peaks in the melting curves (data not shown) confirmed the uniqueness of PCR products. The absolute expression of both miRNAs and the nuclear RNA U6 in bovine oocytes and embryos varied considerably, even between samples from similar developmental stages. To exclude the possibility that the differences in expression levels were due to differences in cDNA synthesis the expression of UniSp6 that was used as a spike-in was examined in a different set of samples that was collected and treated exactly the same as the experimental samples. The levels of UniSp6 did not change throughout the stages analysed indicating similar cDNA synthesis efficiency (Additional file 1: Figure S1). Among the miRNAs tested for bovine oocytes and embryos, miR-93 and miR-103 showed similar and more stable expression patterns. In porcine samples, the average E and R^2^ values were 97.4 % (±10.9) and 0.989 (±0.010) respectively. In these samples, expression of miR-26a, miR-191, miR-93 and miR-103 were most similar.

The absolute expression levels of most miRNAs were highest in bovine morula and blastocyst samples (Additional file 2: Figure S2) and porcine COC samples (Additional file 3: Figure S3). This is most likely caused by higher total RNA levels in those samples.

### Expression stability

To find the optimal set of reference miRNAs in each species, miRNA expression was analyzed using the software packages geNorm and BestKeeper.

**Fig. 1:**
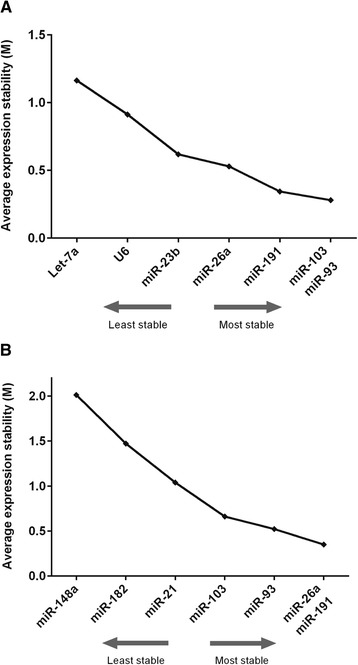
Average expression stability. The expression stability of candidate reference miRNAs as calculated using geNorm. **a** bovine miRNAs, **b** porcine miRNAs. The most stable miRNAs have the lowest expression stability, M

Using geNorm, the average expression stability (M) for the miRNAs was calculated by stepwise exclusion of the miRNA with the lowest expression stability. High variation in expression elevates M values and indicates low stability, whereas low M values indicate high expression stability. In cattle oocytes and embryos, miR-103 and miR-93 showed the lowest M value, and thus highest stability, whereas Let-7a and U6 expression was more variable (Fig. 1a). In porcine samples, miR-26a and miR-191 had the lowest M values, followed by miR-93 (Fig. 1b).

Using BestKeeper, pairwise correlation of the expression of miRNAs was analyzed via raw quantification cycle values. The geometric mean of the best combination of candidates was calculated to establish a BestKeeper index. In cattle, the BestKeeper outcome indicated expression levels of miR-93, miR-103 and miR-191 as having the highest coefficient of correlation [r] indicator of a linear relationship between two variables (Table 1). These miRNAs also demonstrated the highest coefficient of determination [r^2^], which is an index of the proportion of the fluctuations of one variable from the other (Table 2). The combination of miR-93 and miR-103 had the highest r and r^2^ values (Table 1). In porcine samples, miR-93, miR-26a, miR-191 and miR-103 had the highest r and r^2^ values (Tables 3, 4).

**Table 1:** Pearson correlation coefficient for bovine data

vs.	miR-23b	miR-26a	miR-93	miR-103	miR-191	Let-7a	U6
miR-26a	0.772	-	-	-	-	-	-
*p*-value	0.001	-	-	-	-	-	-
miR-93	0.837	0.852	-	-	-	-	-
*p*-value	0.001	0.001	-	-	-	-	-
miR-103	0.805	0.872	0.981	-	-	-	-
*p*-value	0.001	0.001	0.001	-	-	-	-
miR-191	0.822	0.793	0.967	0.963	-	-	-
*p*-value	0.001	0.000	0.001	0.001	-	-	-
Let-7a	0.317	0.330	0.213	0.213	0.185	-	-
*p*-value	0.131	0.115	0.319	0.319	0.388	-	-
U6	0.252	0.258	0.411	0.410	0.428	0.140	-
*p*-value	0.235	0.224	0.046	0.046	0.037	0.516	-
BestKeeper vs.	miR-23b	miR-26a	miR-93	miR-103	miR-191	Let-7a	U6
coeff. of corr. [r]	0.841	0.861	0.947	0.945	0.932	0.412	0.582
*p*-value	0.001	0.001	0.001	0.001	0.001	0.046	0.003

**Table 2:** Regression analysis

	miR-23b	miR-26a	miR-93	miR-103	miR-191	Let-7a	U6
	vs.	vs.	vs.	vs.	vs.	vs.	vs.
	BK	BK	BK	BK	BK	BK	BK
coeff. of corr. [r]	0.841	0.861	0.947	0.945	0.932	0.412	0.582
coeff. of det. [r^2]	0.707	0.741	0.897	0.893	0.869	0.170	0.339
intercept [CP]	6.523	1.629	−6.823	−5.029	−8.145	14.409	−0.709
slope [CP]	0.838	0.960	1.299	1.163	1.353	0.559	0.802
SE [CP]	±0.623	±0.655	±0.508	±0.464	±0.607	±1.428	±1.293
*p*-value	0.001	0.001	0.001	0.001	0.001	0.046	0.003
Power [x-fold]	1.680	1.787	2.048	1.890	1.968	1.520	1.688

**Table 3:** Pearson correlation coefficient for porcine data

vs.	miR-21	miR-26a	miR-93	miR-103	miR-148a	miR-182	miR-191
miR-26a	0.930	-	-	-	-	-	-
*p*-value	0.001	-	-	-	-	-	-
miR-93	0.909	0.984	-	-	-	-	-
*p*-value	0.001	0.001	-	-	-	-	-
miR-103	0.880	0.973	0.995	-	-	-	-
*p*-value	0.001	0.001	0.001	-	-	-	-
miR-148a	−0.407	−0.323	−0.206	−0.180	-	-	-
*p*-value	0.189	0.306	0.518	0.575	-	-	-
miR-182	0.860	0.904	0.868	0.879	−0.506	-	-
*p*-value	0.001	0.001	0.001	0.001	0.093	-	-
miR-191	0.898	0.993	0.986	0.982	−0.311	0.902	-
*p*-value	0.001	0.001	0.001	0.001	0.327	0.001	-
BestKeeper vs.	miR-21	miR-26a	miR-93	miR-103	miR-148	miR-182	miR-191
coeff. of corr. [r]	0.934	0.993	0.996	0.989	−0.258	0.899	0.989
*p*-value	0.001	0.001	0.001	0.001	0.421	0.001	0.001

**Table 4:** Regression analysis

	miR-21	miR-26a	miR-93	miR-103	miR-148a	miR-182	miR-191
	vs.	vs.	vs.	vs.	vs.	vs.	vs.
	BK	BK	BK	BK	BK	BK	BK
coeff. of corr. [r]	0.934	0.993	0.996	0.989	−0.258	0.899	0.989
coeff. of det. [r^2]	0.872	0.986	0.992	0.978	0.067	0.808	0.978
intercept [CP]	−5.322	−8.178	−7.878	−11.251	31.819	19.176	−12.600
slope [CP]	1.070	1.242	1.326	1.405	−0.095	0.435	1.441
SE [CP]	±1.111	±0.401	±0.323	±0.571	±0.96	±0.575	±0.585
*p*-value	0.001	0.001	0.001	0.001	0.421	0.001	0.001
Power [x-fold]	1.717	2.516	2.624	2.997	0.933	1.310	2.094

### Optimal number of reference miRNAs

**Fig. 2:**
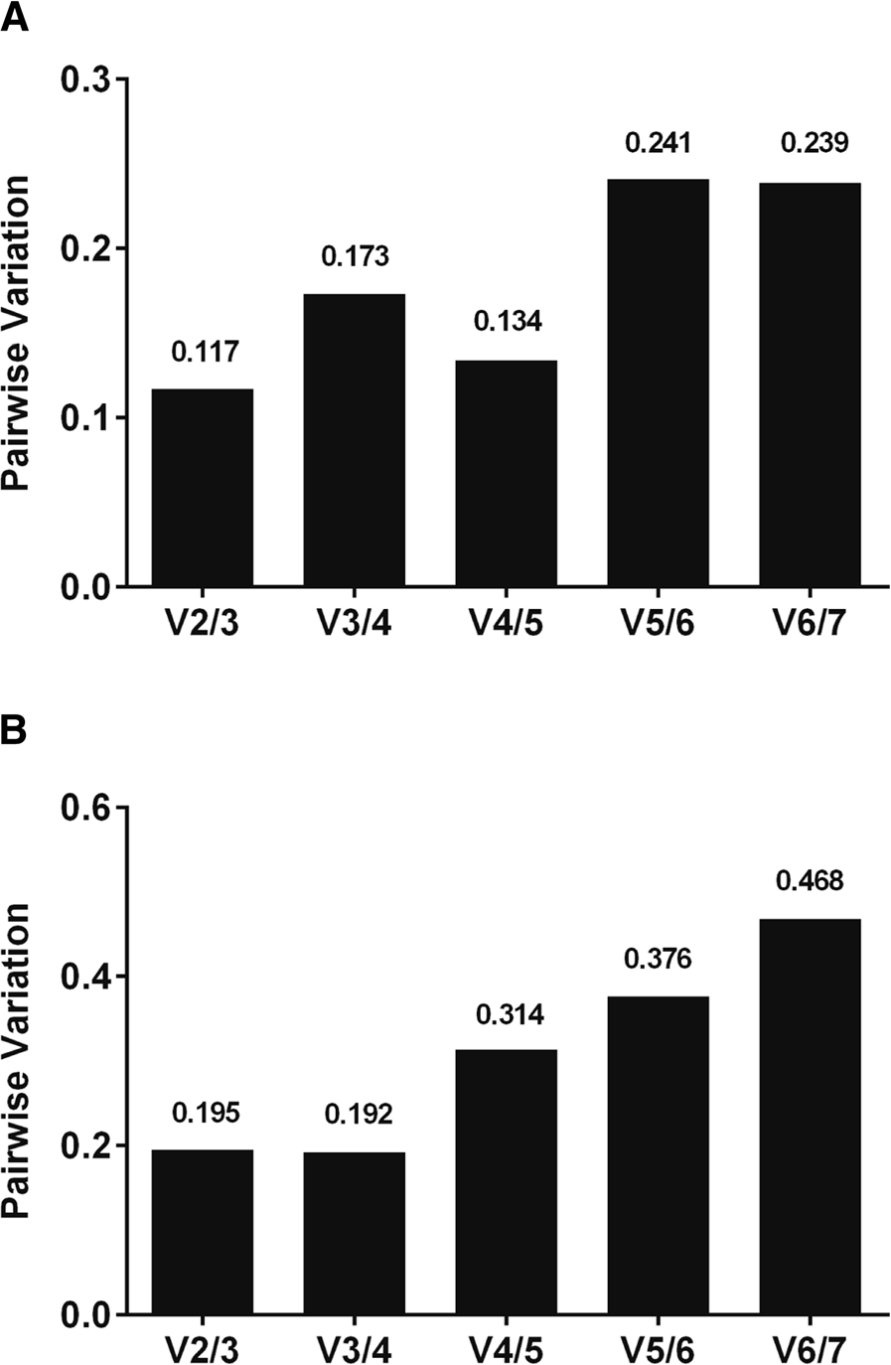
Optimal number of reference miRNAs. Pairwise variation after successive inclusion of candidate reference miRNAs with lower stability using geNorm provided the optimal combination of miRNAs for normalization purposes. **a** In bovine oocytes and embryos, the use of two miRNAs was optimal. **b** In porcine oocytes and COCs the combination of three miRNAs was optimal, although the difference between using 2 or 3 miRNAs was negligible

To identify the optimum number of reference miRNAs, stepwise inclusion of the examined miRNAs was assessed. In the bovine samples, inclusion of the two most stable reference miRNAs yielded less variation than other combinations, and including more miRNAs did not improve the normalization factor (Fig. 2a). In porcine oocytes, the combination of three candidates showed the highest stability, although the difference with the use of 2 reference miRNAs was negligible (Fig. 2b).

**Fig. 3:**
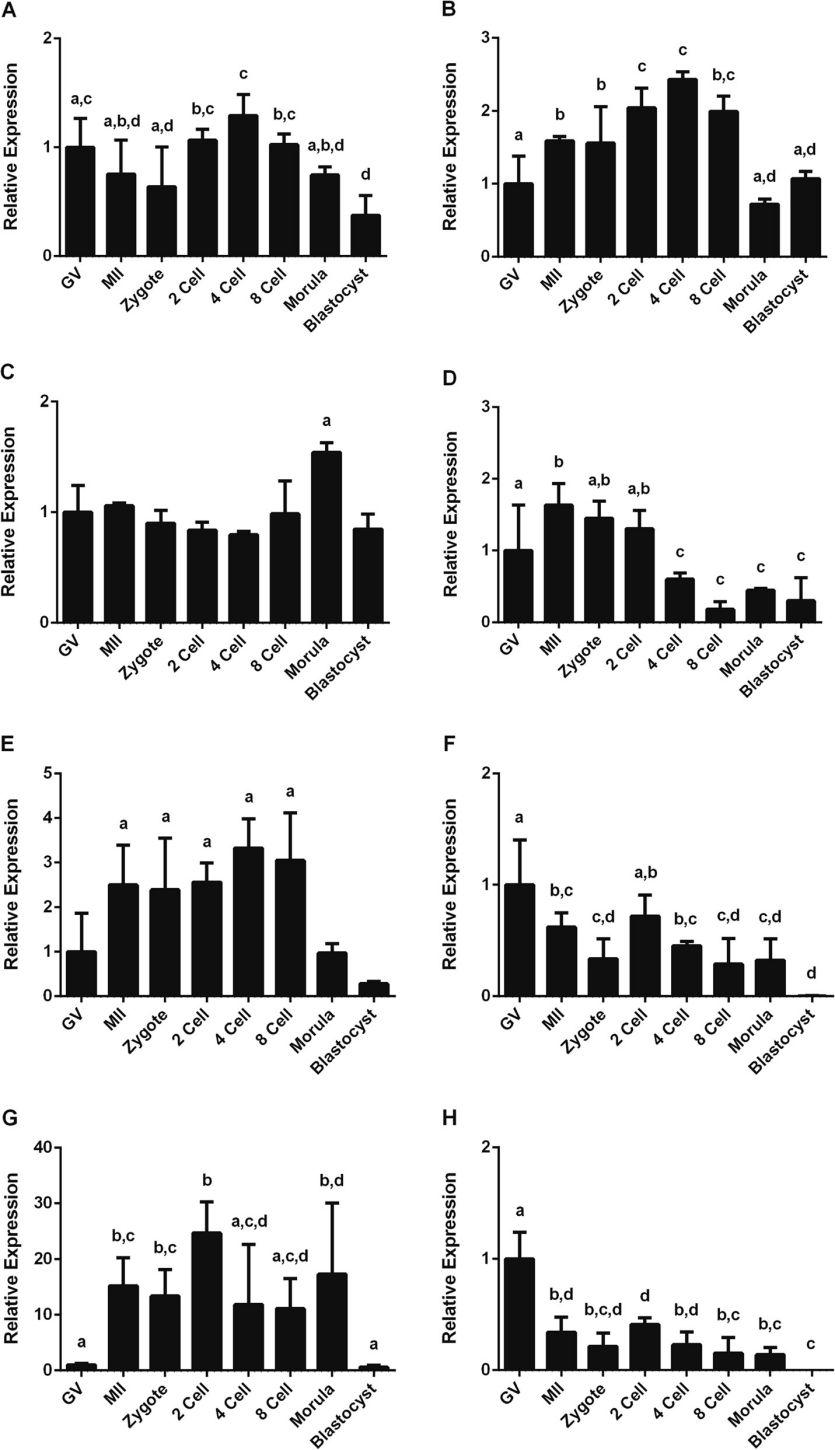
Relative expression of bovine miRNAs after normalization with miR-93 and miR-103. (**a**) miR-23b, (**b**) miR-26a, (**c**) miR-191, (**d**) U6, (**e**) Let-7, (**f**) miR-Let-7b, (**g**) miR-222, (**h**) miR-224. Different letters above bars (*a,b,c,d*) indicate values that differ significantly (*p* < 0.05)

**Fig. 4:**
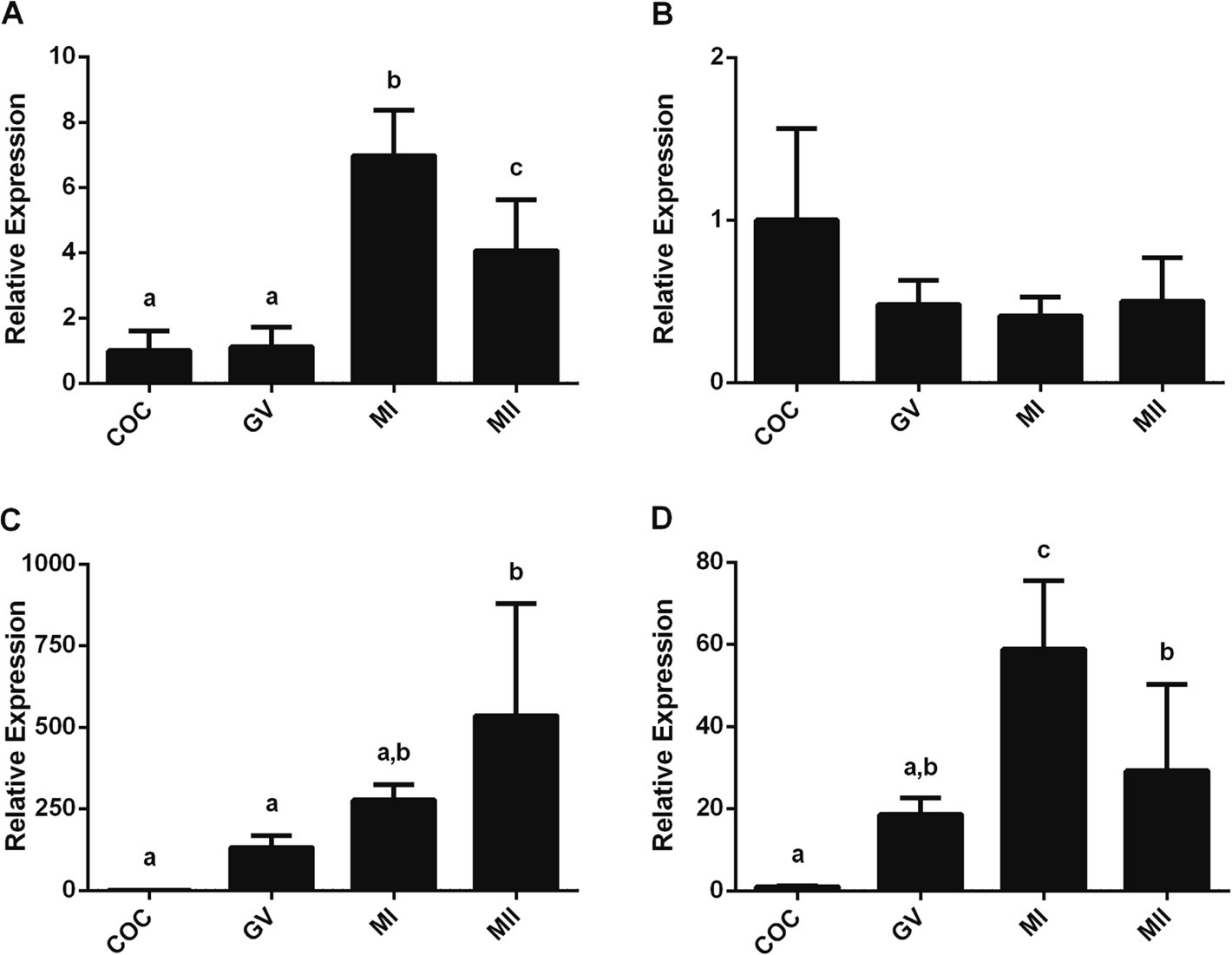
Relative expression of porcine miRNAs after normalization with miR-26a, miR-191 and miR-93. (**a**) miR-21, (**b**) miR-103, (**c**) miR-148a, (**d**) miR-182. Different letters above bars (*a,b*) indicate values that differ significantly (*p* < 0.05)

Expression of the miRNAs miR-93 and miR-103 was subsequently used to normalize the expression levels of the other miRNAs and nuclear RNA analyzed in bovine samples (Fig. 3a-e). In porcine samples, normalization was performed using the relative expression of miR-26a, miR-191 and miR-93 (Fig. 4).

In bovine oocytes and embryos, all examined miRNAs showed an average expression stability below 1.5 (Fig. 1a) indicating relatively stable expression. Using the geometric mean of the identified reference miRNAs for normalization we therefore examined the expression of other miRNAs in oocytes and early embryos, namely Let-7b, miR-222 and miR-224 (Fig. 3f-h). Let-7b is a member of the Let-7 family of miRNAs shown to affect cell cycle molecules [39]. miR-222 was reported to be enriched in bovine mature oocytes and cumulus cells [22] and miR-224 has been shown to mediate the stimulatory effects of TGFβ1 on mouse granulosa cell proliferation by targeting Smad4, which is an important player in signal transduction of the TGFβ superfamily [40].

The 3 new miRNAs showed different expression patterns to the original panel examined. Let-7b was expressed at highest levels in GV stage oocytes and was absent in blastocyst stage embryos (Fig. 3f). By contrast, expression of miR-222 was almost absent in GV stage oocytes but had significantly increased by the MII stage (Fig. 3g). The expression of miR-224 followed a pattern similar to that of Let 7b (Fig. 3h).

## Discussion

When using qRT-PCR to compare gene expression levels, normalization using genes for which the expression does not vary appreciably between the cell types or stages is essential. Indeed, it is generally advised to normalize data acquired by qRT-PCR by using expression levels of more than one of these so-called reference genes [25, 41]. Correct normalization is essential to rectify for differences in the efficiency of RNA isolation between samples, levels of RNA degradation, efficiency of cDNA synthesis, amount of material processed and simple experimental variation. Historically, normalization of mRNA expression was performed with only one reference gene, often *GAPDH*. It has become clear, however, that for accurate normalization the use of several internal control genes is more reliable, and software such as geNorm and BestKeeper have been developed to help identify genes with the most stable expression levels.

The expression of reference genes can vary between different tissue types and the ideal reference genes should have constant expression levels in the tissue of interest irrespective of the experimental conditions. For analyzing the expression of miRNA in cells and tissues, however, the use of reference genes is likely to be suboptimal. There are several potential disadvantages of using genes (mRNA) or other nuclear RNAs for normalization of miRNA data. Firstly, these two groups (miRNAs vs mRNA/nuclear RNAs) have different pathways of biogenesis and are, by definition, different in nature. Secondly, there are significant size differences between miRNA and mRNA molecules which affects the methods and efficiency of RNA isolation and cDNA synthesis [38].

In contrast with methods for normalizing mRNA expression, little is known about the reliability of mRNA or miRNA for normalizing miRNA expression data. Our strategy was to select several potential candidates and examine their expression patterns using qRT-PCR, before using geNorm and BestKeeper to identify sets of miRNAs with the least variation for subsequent use as references for normalization.

There are various methods for normalization of expression levels. Expression levels can be calculated by relating the Ct values to each other, known as the 2^-ΔΔCt^ method [42, 43]. Alternatively, the global mean of all expressed miRNAs can be considered as a normalizing factor [36, 37]. Here we incorporated amplification efficiency since relative expression was calculated using specific standard curves.

In the present study, the stability of expression of several miRNAs and a nuclear RNA, U6, previously reported for normalization of miRNA expression was tested in bovine and porcine oocytes and embryos. Using the geNorm conventions, we considered that expression could be considered ‘stable’ when the average expression stability was less than 1.5. In this study, all candidate miRNAs could thus be considered suitable for normalization of miRNA expression in bovine oocytes and preimplantation embryos (M values ranged from 0.28 to 1.1). In porcine COCs and oocytes only miR-184 did not meet the criteria for stability (M value was 2.01). Among the different miRNAs however, miR-93 and miR-103, followed by miR-191, in bovine samples and miR-26a, miR-191 and miR-93 in porcine oocytes had the least variation and are therefore considered the most suitable for normalization. The expression of miR-21 in porcine cells was similar to that described previously with an increase in expression levels during maturation [44]

Here we have identified miRNAs that are expressed at a relatively constant level throughout normal oocyte and early embryo development. Abnormal or unstable expression of these miRNAs could indicate abnormal development. Although individual miRNA species can target multiple genes we had anticipated that the miRNAs that we had identified as reference would be more directed at genes in involved in basic cell biology. In silico analysis of the targets of the miRNAs suitable as reference versus those less suited did not reveal any obvious differences however, nor pointed at specific ‘houskeeping’ targets for the reference miRNAs (not shown).

Interestingly, miR-93 was reported to be a stable miRNA in human and porcine normal and cancerous solid tissues [32, 35]. In addition, the combination of miR-93 and miR-16 was found to be suitable for normalizing miRNA expression in the serum of gastric cancer patients and healthy controls [34]. miR-103 was proposed to be a stable miRNA in the porcine liver and uterus, but the least stable in the ovary [32], whereas it was considered unstable in rat tissues [45]. miR-191 was proposed as the best (most stable) miRNA data normalizer in the serum of human colorectal adenocarcinoma and colorectal adenoma patients [33], but had a lower stability ranking in rat tissues [45].

U6 is the major spliceosomal small nuclear RNA involved in processing of pre-mRNA [46] and although not a miRNA it is commonly used for normalization of miRNA expression [42, 43, 47]. We found expression of U6 to be unsuitable for normalization of miRNA expression in bovine oocytes and preimplantation embryos as its expression gradually decreased during the early cleavage stages. As there is little or no transcription before embryonic genome activation around the 8 cell stage this might reflect degradation of maternal RNA. This dynamics in U6 expression was similar to what has been described [19] We also found Let-7a to be the least stable miRNA in bovine oocytes and early embryos. This parallels reports in rat tissues and human serum where Let-7a expression was less stable than that of other candidates [34, 45].

## Conclusion

Our results demonstrate interspecies variation in most suitable reference miRNAs and indicate the necessity of examining several potential miRNAs in different experimental models. The identification of a set of constantly expressed miRNAs will help us to better understand the function of miRNAs in the oocytes and preimplantation embryos.

## Additional files

Additional file 1: Figure S1. Expression of spiked-in UniSp6 added to RNA of the indicated cells and embryo stages. The expression level is plotted as threshold cycle (Ct). Additional file 2: Figure S2. Absolute expression of candidate miRNAs in bovine oocytes and embryos. (A) miR-23b, (B) miR-26a, (C) miR-93, (D) miR103, (E) miR-191, (F) Let-7a, (G) U6. Additional file 3: Figure S3. Absolute expression of candidate miRNAs in porcine oocytes. Left (1) graphs show all groups with the expression of cumulus-oocyte complexes (COC) set at 1; right (2) graphs show the same but the expression in germinal vesicle (GV) oocytes set at 1 and without COCs. (A) miR-21, (B) miR-26a, (C) miR-93, (D) miR-103, (E) miR-148a, (F) miR-182, (G) miR-191.
