# Supplementary figures and images for: Validating reference microRNAs for normalizing qRT-PCR data in bovine oocytes and preimplantation embryos
Source: BMC Dev Biol. 2015 Jun 12;15:25. doi: 10.1186/s12861-015-0075-8 (PMC4464232; doi:10.1186/s12861-015-0075-8)

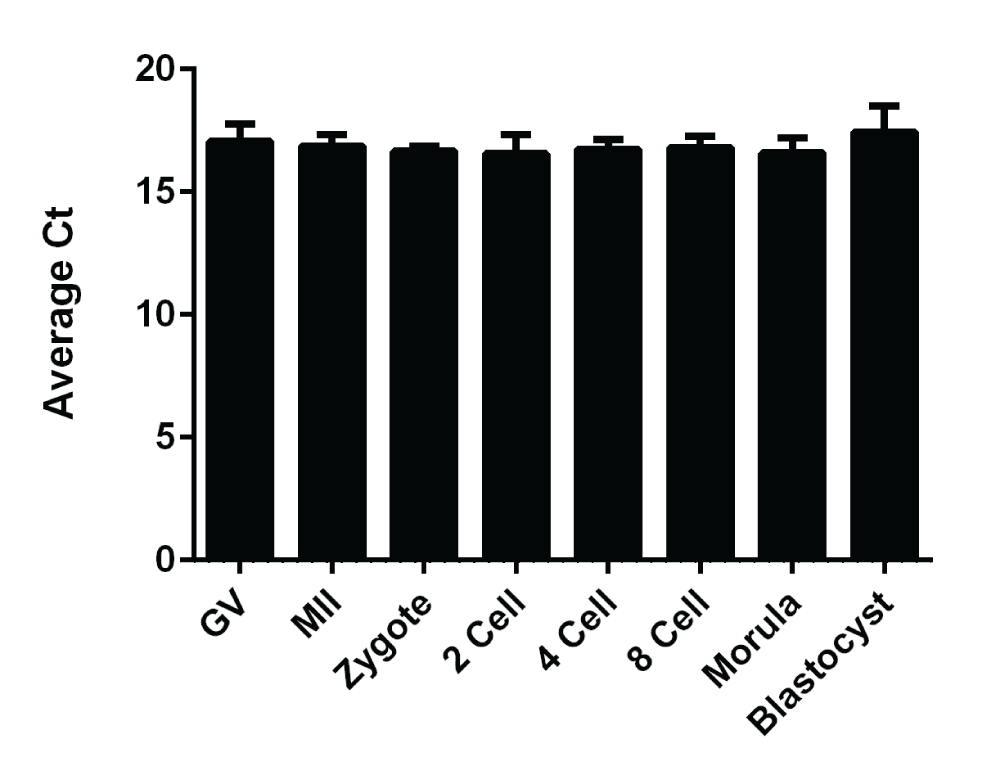

Supplement: Additional file 1: Figure S1. — Expression of spiked-in UniSp6 added to RNA of the indicated cells and embryo stages. The expression level is plotted as threshold cycle (Ct). [file 12861_2015_75_MOESM1_ESM.tif]

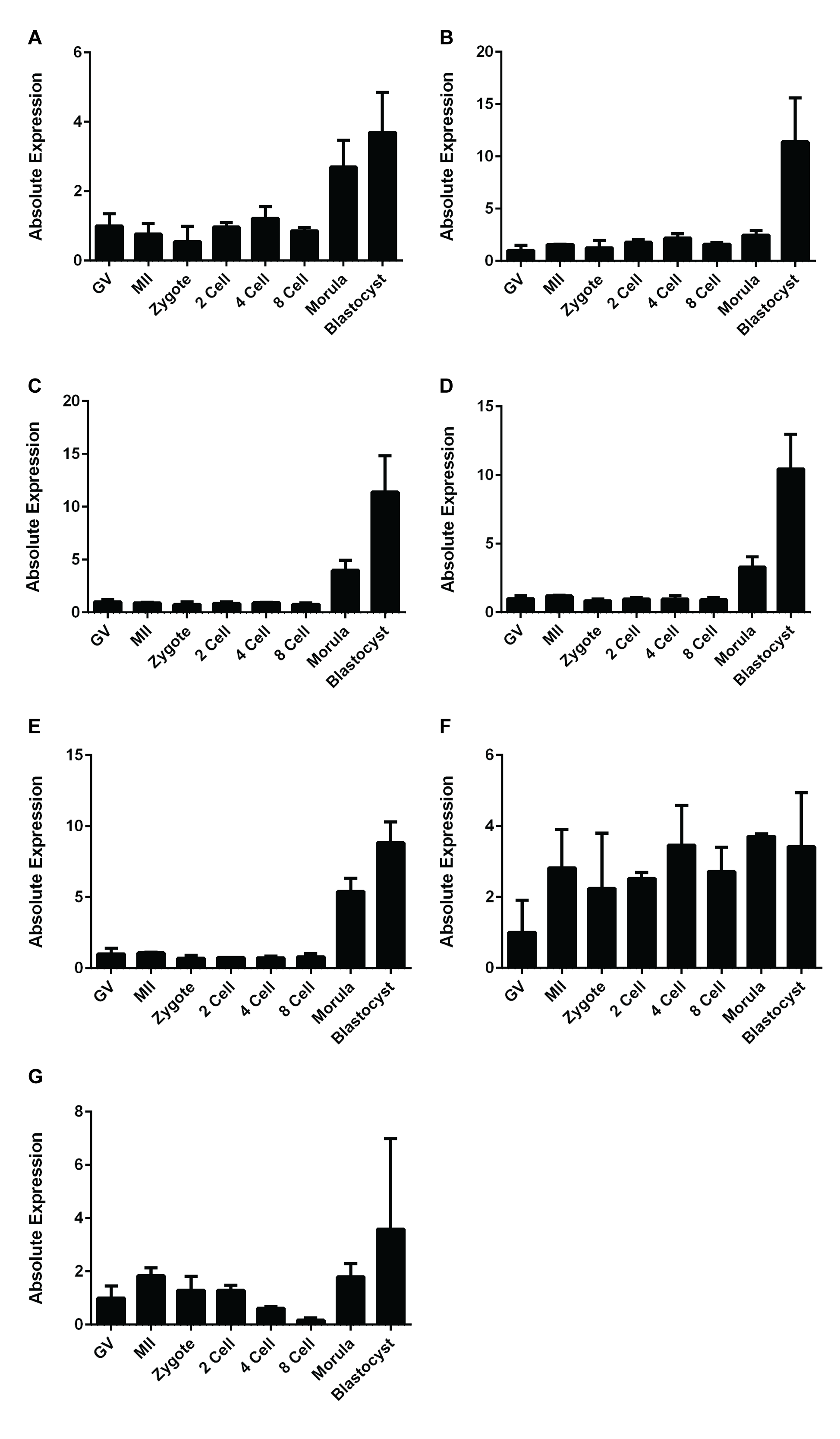

Supplement: Additional file 2: Figure S2. — Absolute expression of candidate miRNAs in bovine oocytes and embryos. (A) miR-23b, (B) miR-26a, (C) miR-93, (D) miR103, (E) miR-191, (F) Let-7a, (G) U6. [file 12861_2015_75_MOESM2_ESM.tif]

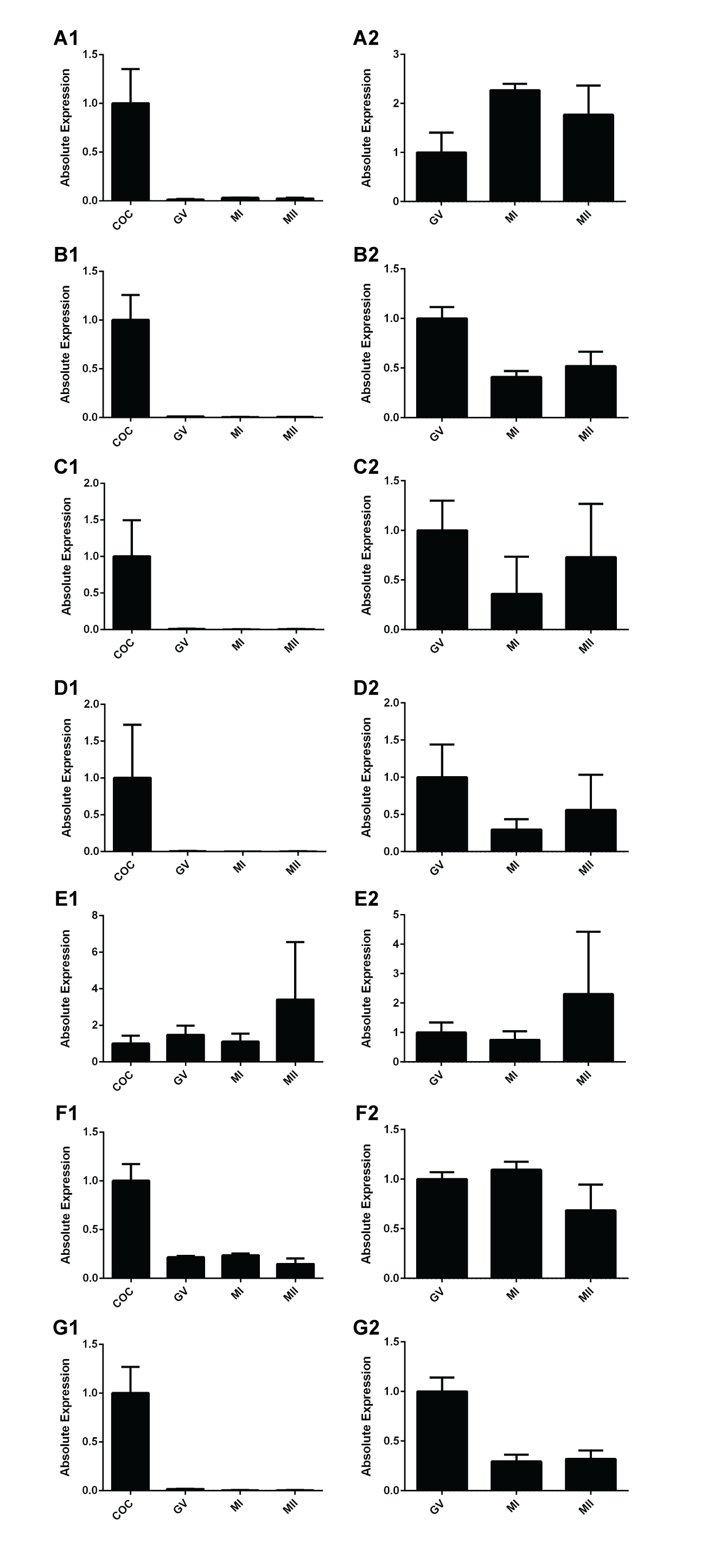

Supplement: Additional file 3: Figure S3. — Absolute expression of candidate miRNAs in porcine oocytes. Left (1) graphs show all groups with the expression of cumulus-oocyte complexes (COC) set at 1; right (2) graphs show the same but the expression in germinal vesicle (GV) oocytes set at 1 and without COCs. (A) miR-21, (B) miR-26a, (C) miR-93, (D) miR-103, (E) miR-148a, (F) miR-182, (G) miR-191. [file 12861_2015_75_MOESM3_ESM.tif]
